# Porter 6: Protein Secondary Structure Prediction by Leveraging Pre-Trained Language Models (PLMs)

**DOI:** 10.3390/ijms26010130

**Published:** 2024-12-27

**Authors:** Wafa Alanazi, Di Meng, Gianluca Pollastri

**Affiliations:** 1 School of Computer Science, University College Dublin (UCD), D04 V1W8 Dublin, Ireland; wafa.alanazi@ucdconnect.ie (W.A.); di.meng@ucdconnect.ie (D.M.); 2 Department of Computer Science, College of Science, Northern Border University, Arar P.O. Box 2014, Saudi Arabia

**Keywords:** protein structure prediction, structural bioinformatics, bioinformatics, natural language processing, computational biology, deep learning

## Abstract

Accurately predicting protein secondary structure (PSSP) is crucial for understanding protein function, which is foundational to advancements in drug development, disease treatment, and biotechnology. Researchers gain critical insights into protein folding and function within cells by predicting protein secondary structures. The advent of deep learning models, capable of processing complex sequence data and identifying meaningful patterns, offer substantial potential to enhance the accuracy and efficiency of protein structure predictions. In particular, recent breakthroughs in deep learning—driven by the integration of natural language processing (NLP) algorithms—have significantly advanced the field of protein research. Inspired by the remarkable success of NLP techniques, this study harnesses the power of pre-trained language models (PLMs) to advance PSSP prediction. We conduct a comprehensive evaluation of various deep learning models trained on distinct sequence embeddings, including one-hot encoding and PLM-based approaches such as ProtTrans and ESM-2, to develop a cutting-edge prediction system optimized for accuracy and computational efficiency. Our proposed model, Porter 6, is an ensemble of CBRNN-based predictors, leveraging the protein language model ESM-2 as input features. Porter 6 achieves outstanding performance on large-scale, independent test sets. On a 2022 test set, the model attains an impressive 86.60% accuracy in three-state (Q3) and 76.43% in eight-state (Q8) classifications. When tested on a more recent 2024 test set, Porter 6 maintains robust performance, achieving 84.56% in Q3 and 74.18% in Q8 classifications. This represents a significant 3% improvement over its predecessor, outperforming or matching state-of-the-art approaches in the field.

## 1. Introduction

Protein secondary structure prediction (PSSP) is a fundamental component of protein structure prediction, crucial for understanding the structural characteristics necessary for inferring a protein’s tertiary structure. Secondary structure refers to the local conformation of the protein’s polypeptide backbone [1]. Linus Pauling’s foundational research recognizes two primary structural motifs: the α-helix (H) and the β-strand (E) [1]. The α-helix forms a helical structure stabilized by hydrogen bonds at every fourth amino acid. At the same time, the β-strand adopts a parallel or antiparallel configuration through hydrogen bonds between amino acids. A third category, the random coil (C), accounts for amino acids that do not conform to the α-helix or β-strand structures [1].

PSSP is a classification task where each amino acid residue is categorized based on its secondary structure. PSSP is typically divided into two classifications: three-state (H, E, C) and eight-state (H, G, I, B, E, S, T, C), with the latter providing a more detailed description of secondary structural elements, including additional motifs like the 3_10_-helix (G), isolated β-bridge (B), bend (S), turn (T), and π-helix (I). These classifications are determined by hydrogen bonding patterns and geometric features, which can be computationally derived from atomic coordinates using tools such as DSSP (define secondary structure of proteins) [2].

The evolution of protein secondary structure prediction (PSSP) methods has progressed through five generations, each marked by increasing complexity and accuracy. Early methods relied on the statistical tendencies of amino acids, yielding limited precision [3]. Later generations introduced advanced statistical techniques such as graph theory and neural networks, which improved prediction accuracy by analyzing residue windows [3]. The third generation incorporated evolutionary information from multiple sequence alignments (MSA) and profile-based inputs like PSSM and HMM, which led to significant accuracy gains [4]. Fourth-generation models further integrated physicochemical properties and deep learning techniques, driving prediction accuracy above 80% [4]. Finally, the fifth generation embraced deep learning architectures, such as convolutional neural networks (CNNs) and recurrent neural networks (RNNs), and combined them with evolutionary and physicochemical data to enhance predictions [4].

Over the past decade, protein structure prediction has seen remarkable progress, largely due to increased computational power and the expansion of protein sequence databases. Traditional PSSP methods often rely on multiple sequence alignments (MSAs), which align homologous sequences to capture evolutionary information [5]. Common profiles, such as the position-specific scoring matrix (PSSM) and hidden Markov model (HMM), typically obtained using tools like PSI-BLAST [6] or HHblits [7], have been instrumental in improving PSSP accuracy. Although these profiles enhance prediction accuracy, the MSA-based embedding technique has limitations, including computational complexity, time consumption, and difficulties capturing diverse contextual information [8]. These challenges reduce its effectiveness, particularly for proteins with few or no homologous sequences.

Deep learning models, particularly CNNs and RNNs, have become central to PSSP research [4]. Notable models from this era include NetSurfP-2.0 (2019) [9], SPOT-1D (2018) [10], and Porter 5 (2019) [5], each contributing incremental improvements to secondary structure prediction. NetSurfP-2.0, for instance, leveraged CNNs and MSAs to predict not only secondary structure but also solvent accessibility and disorder. SPOT-1D enhanced prediction accuracy using an ensemble of neural networks and sequence alignment profiles.

Recently, the success of language models (LMs) in natural language processing has sparked interest in applying these models to protein structure prediction. Protein language models (PLMs), trained on vast protein databases like Uniref23, Uniclust24, Pfam25, and BFD26, can derive embedding features that capture contextual information about amino acid residues. In this context, these PLMs, such as ProtT5-XL-U50 [11] and ESM-2 [12], are emerging as potential replacements for evolutionary information in PSSP due to their ability to generate information-rich representations of protein sequences, enabling more accurate secondary structure predictions [4]. Additionally, PLM pre-training on extensive protein sequence data has shown promise in protein-related downstream tasks, including protein structure prediction, subcellular localization prediction, and membrane protein prediction [2]. SPOT-1D-LM [13] and NetSurfP-3.0 represent recent advances in PSSP, utilizing PLM embeddings rather than MSAs. SPOT-1D-LM employs ensemble learning by training models on embeddings from ProtT5-XL-U50 and ESM-1b, achieving comparable performance to traditional MSA-based methods. Similarly, NetSurfP-3.0 replaces MSAs with language model embeddings (ESM-1b), dramatically reducing runtime while maintaining high accuracy.

Recent technological advancements, particularly in deep learning, have revolutionized protein structure prediction, leading to the development of state-of-the-art methods like AlphaFold2 [14]. These methods have surpassed traditional approaches that relied solely on single protein sequences or structural models. However, despite these advancements, AlphaFold2′s reliance on strong sequence homology and MSA limits its application [15]. Furthermore, its high computational demands make it impractical for some applications. As a result, there is a continued need for more efficient and accurate PSSP models, particularly for proteins without known homologs.

Protein secondary structure prediction has been a core focus of bioinformatics for decades, yet even the most sophisticated ab initio SS predictors cannot achieve the theoretical limit of three-state prediction accuracy (~94%) [16]. These challenges highlight the need for further research to develop more efficient and accurate predictive models. Thus, this study addresses these challenges by systematically evaluating various deep learning models and their combinations to determine the most effective approach for PSSP. In particular, we focus on leveraging pre-trained protein language models (PLMs) to generate information-rich embeddings that capture complex relationships within protein sequences. The main contributions of this study include the following:

1. A comprehensive evaluation of different deep learning models, including CNNs, LSTM, RNNs, and CBRNN, to identify the most effective strategy for PSSP.
2. A comparison of encoding methods, including one-hot, ProtTrans, and ESM-2, to determine the optimal approach for protein sequence representation.
3. The utilization of large training datasets, beginning with half a million proteins, and the implementation of redundancy reduction protocols (at 80% and 30% thresholds) to optimize model performance across benchmark datasets.

This paper advances the state-of-the-art in PSSP by addressing existing limitations, improving predictive accuracy, and reducing computational overhead, ultimately contributing to more effective methods for protein structure prediction.

## 2. Results

The evaluation process for our protein secondary structure prediction model was conducted in three phases:

1. Phase 1: Evaluation of various embedding methods, model architectures, and their combinations.
2. Phase 2: Implementation of the best combinations to predict secondary structures in three-state and eight-state classifications, along with testing different training strategies.
3. Phase 3: The optimal configurations are applied to a larger dataset to develop final predictors, which are then evaluated using the benchmark 2024 test set to ensure accuracy and robustness.

### 2.1. Phase 1: Evaluation of Embedding Methods and Model Architectures

#### 2.1.1. Performance of Baseline Embedding Methods

The first phase began with a baseline model architecture, utilizing a simple feedforward neural network (FFNN) to evaluate various embedding methods, including one-hot encoding, ProtTrans, and ESM-2. As shown in Table 1, the results demonstrate that PLM-based embedding methods consistently outperform one-hot encoding. Among the PLM-based embeddings, ESM-2 demonstrated slightly superior performance, achieving an accuracy ACC (Q3) of 85.68%.

#### 2.1.2. Performance Across Advanced Model Architectures

Advanced architectures, including convolutional neural networks (CNNs), recurrent neural networks (RNNs), and long short-term memory (LSTM) networks, were evaluated for their compatibility with different embedding methods.

We explored increasingly complex model architectures, beginning with convolutional neural networks (CNNs). Hyperparameter tuning started with a single-layer configuration, gradually increasing kernel size, number of channels, and layers until performance improvements plateaued. The CNN with the 11-layer architecture consistently delivered the best results among all embedding methods.

We also evaluated recurrent neural networks (RNNs) and long short-term memory (LSTM) networks. Despite rigorous hyperparameter tuning, RNNs and LSTMs could not match the performance of the CNN_L11 model across all embedding methods. CNN models, particularly the CNN_L11 with ESM-2 embeddings, achieved superior global representation, with the highest ACC3 score of 86.20%.

The convolutional bidirectional recurrent neural network (CBRNN) model, which integrates both RNN and CNN components with predefined hyperparameters, produced slightly better results than standalone RNNs and LSTMs. Using ESM-2, the CBRNN achieved the highest performance with an ACC3 of 86.34%. Given its superior performance, CBRNN was selected as the preferred architecture for further training in subsequent phases. Furthermore, Table 2 presents the results from Phase 1, evaluating embedding methods and model architectures across predictors.

### 2.2. Phase 2: Implementation of Best-Embedded Sequences and Best Model

#### 2.2.1. Performance in Three-State and Eight-State Predictions

From Phase 1, the CBRNN model architecture and ESM-2 embeddings emerged as the most effective for three-class secondary structure predictions (helix, strand, and coil). In this second phase, we aimed to evaluate the performance of this combination on eight-class predictions using the 2022 test sets while training on the PDB: 30% dataset. The model achieved an ACC (Q3) of 86.34% for three-state predictions and ACC (Q8) of 75.23% for eight-state predictions, as shown in Table 3.

#### 2.2.2. Training Strategies

We tested two distinct training strategies: full-set training and five-fold cross-validation. The full-set training involved using the entire dataset to train the model for both three-class and eight-class predictions. In contrast, the five-fold cross-validation approach involved dividing the dataset into five parts, training the model on four parts while testing on the fifth, and rotating through all parts. The five-fold cross-validation strategy was tested with ESM-2 embeddings for three-class predictions and ESM-2 embeddings for eight-class predictions. This approach slightly outperformed the full-set training strategy, achieving 86.60% for three-class predictions and 75.43% for eight-class predictions, as summarized in Table 4.

The detailed performance across the different folds during the five-fold cross-validation is summarized in Table 5. The five-fold cross-validation strategy demonstrated slightly better performance and more consistency compared to full-set training. As a result, this strategy will be carried forward into Phase 3 for further model development and evaluation.

### 2.3. Phase 3: Larger Dataset and Ensemble Predictors

#### 2.3.1. Performance on Larger Dataset

This study was expanded by shifting to a larger dataset, PDB: 80%, and fine-tuning the CBRNN model architecture to optimize performance. Through our observations, we found that increasing the depth of the CNN in the CBRNN model from two to three layers (CBRNN_L3) yielded optimal performance when predicting three classes. Similarly, for eight-class predictions, performance improved when the depth of the CNN was increased from three to four layers.

Additionally, we conducted a five-fold cross-validation using two clustered datasets: PDB: 80% and PDB: 30%. For each fold, the entire PDB: 80% dataset served as the training set, while one of the five parts of the PDB: 30% dataset was designated as the test set. Before training, the sequences in the PDB: 80% dataset were filtered to remove any sequences exceeding 1022 amino acids in length.

The model was then trained using the filtered PDB: 80% dataset and evaluated on each fold’s respective PDB: 30% as test set. The results of the five-fold cross-validation are summarized in Table 6. The model demonstrated consistent performance across all folds, with an average ACC3 of 86.46% and an average ACC8 of 75.10%. These results indicate that the model can generalize well across different subsets of the dataset, providing reliable predictions for protein secondary structures.

#### 2.3.2. Ensemble and Final Predictors

Our final prediction is Porter 6 derived from Phase 3 based on an ensemble of CBRNN architecture trained on a PDB: 80% 55k proteins. This represents the largest training set used to the best of our knowledge. Porter 6 consists of five predictors for ACC3 and five predictors for ACC8. Each of the five predictors are generated from the five-fold cross-validation process. This ensemble approach ensures superior accuracy and robustness, positioning Porter 6 as a state-of-the-art tool for protein secondary structure prediction.

#### 2.3.3. Comparer Performance

We analyzed the performance of Porter 6 separately on proteins resolved by nuclear magnetic resonance (NMR) and X-ray crystallography. The results for each subset are presented in Table 7. Porter 6 demonstrated robust performance across both subsets, with slightly higher accuracy observed for X-ray-resolved proteins. This is likely due to the higher confidence in structural annotations derived from X-ray crystallography. Conversely, NMR proteins were predicted with slightly lower accuracy, which may be attributed to differences in dataset characteristics, such as shorter average sequence lengths and greater structural flexibility, or less certain secondary structure determinations inherent in NMR data.

We also evaluated Porter 6 against Porter 5 (2019) [5], NetSurfP-2.0 (2019) [9], NetSurfP 3.0 (2022) [8], and SPOT-1D-LM (2022) [13] using 2024 the benchmark test set. This benchmark dataset, consisting of 692 proteins, was used to assess the models based on their Q3 and Q8 accuracies.

The results demonstrate that Porter 6 achieves the highest overall performance, with a Q3 accuracy of 84.56% and a Q8 accuracy of 74.18%, surpassing all other models tested. This represents a significant improvement over its predecessor, Porter 5, which achieved Q3 and Q8 accuracies of 81.03% and 70.01%, respectively. Similarly, Porter 6 outperforms NetSurfP-2.0 (Q3: 81.37%; Q8: 70.06%) and NetSurfP-3.0 (Q3: 82.92%; Q8: 71.84%) the latter showing an improvement over its earlier version but still falling short of Porter 6. SPOT-1D-LM, while competitive with a Q3 accuracy of 84.30% and a Q8 accuracy of 74.09%, does not surpass Porter 6. These results highlight the advancements made by Porter 6 in secondary structure prediction, particularly in improving both Q3 and Q8 metrics as detailed in Table 8.

This evaluation underscores the progress achieved with Porter 6, particularly in its ability to deliver high accuracy without relying on sequence alignments. Porter 6 has demonstrated that it can match or exceed the performance of traditional sequence profile-based prediction methods, which typically require sequence alignments to capture evolutionary information by using leverage protein language models (PLMs) instead of alignments.

## 3. Discussion

This study demonstrates substantial advancements in protein secondary structure prediction (PSSP) by leveraging deep learning techniques and modern protein language models (PLMs). Through systematic evaluation of various embedding methods and neural network architectures, the convolutional bidirectional recurrent neural network (CBRNN) with ESM-2 embeddings emerged as the most effective configuration, achieving high accuracy in both three-state (Q3) and eight-state (Q8) classifications.

The integration of PLMs in this study, specifically ESM-2, was a key driver of success. These embeddings offer rich, context-aware representations that capture local and global sequence information, enabling better secondary structure predictions. Moreover, the use of CNN layers, particularly with deeper architectures, allowed for the extraction of more complex features, further improving the model’s performance.

Another significant contribution of this study is the application of different redundancy reduction thresholds. When trained using an 80% identity threshold, the results showed improved accuracy compared to the dataset filtered with a 30% redundancy reduction. Specifically, Porter 6 achieved higher Q3 and Q8 accuracy scores at the 80% threshold (84.56% and 74.18%, respectively) compared to the 30% redundancy reduction dataset (84.40% and 74.01%, respectively). This indicates that the higher identity threshold enhances model performance in predicting secondary structure.

The elimination of multiple sequence alignments (MSAs) through PLM embeddings is another significant advancement. By bypassing the MSA step, the model not only reduces computational complexity but also achieves superior scalability without sacrificing predictive accuracy. This approach allows for faster predictions, making it highly suitable for large scale applications in protein structure prediction.

AlphaFold2 [14] has revolutionized protein structure prediction by achieving unprecedented accuracy in 3D modelling. However, its reliance on multiple sequence alignments (MSAs) and strong sequence homology requiring at least 30 effective homologous sequences to achieve accurate structure prediction [13] limit its applicability for proteins without known homologs. Additionally, its high computational demands make it less suitable for high-throughput applications. In contrast, Porter 6 leverages protein language model embeddings, enabling accurate secondary structure prediction without the need for MSAs.

## 4. Materials and Methods

### 4.1. Dataset

The selection and preparation of datasets are critical in machine learning tasks, especially in protein secondary structure prediction (PSSP) [17]. For this study, we constructed our datasets using the Protein Data Bank (PDB) [18], a publicly accessible repository of protein structural data. We started with a comprehensive dataset comprising 500,624 protein sequences from PDB entries released up to 16 November 2022. These include PDB: 30% and PDB: 80%, derived through sequence identity clustering. Also, to ensure the integrity of the protein structures, chain breaks were identified through analysis of Cα atom distances exceeding 4.6 Å and subsequently removed during preprocessing.

**Figure 1:**
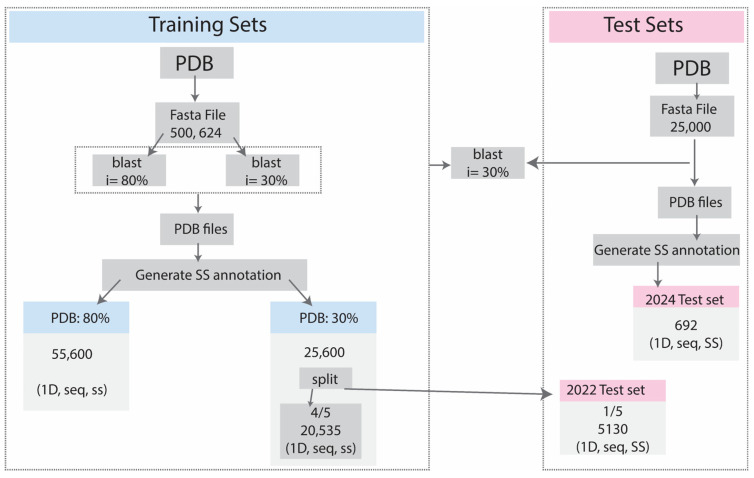
The data collection and dataset preparation process for training and test sets.

**Figure 2:**
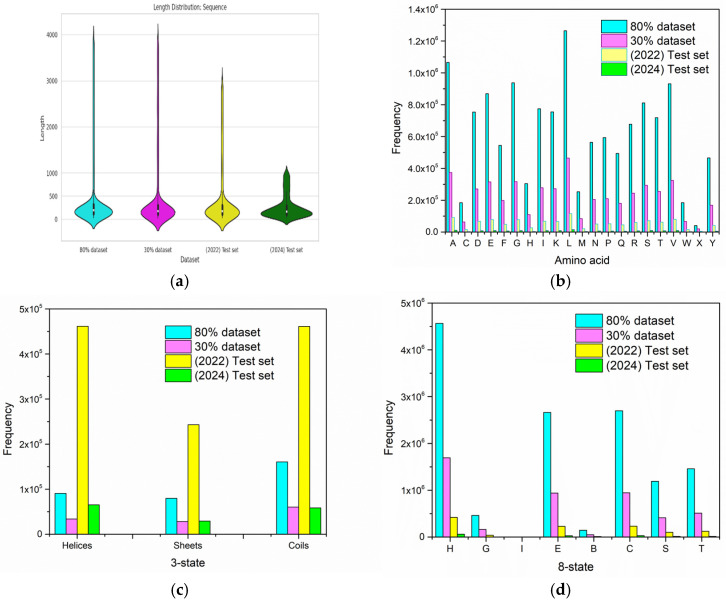
Dataset representation. The charts present a basic analysis of four datasets: 80%, 30%, 2022 test set, 2024 test set: (**a**) Violin plots for sequence length distribution; (**b**) Length distribution for (AA) sequence; (**c**) overview of SS frequency in 3-state classifications; (**d**) overview of SS frequency in 8-state classifications.

For primary testing, we split an initial test set from PDB: 30%, and for the final evaluation, we built an independent benchmark test set from PDB entries in the 2024 test set, consisting of 692 proteins. The detailed dataset collection and overview of the datasets are shown in Figure 1 and Table 9. Additionally, the dataset representation is visualized in Figure 1, which includes violin plots of sequence length distributions (Figure 2a); it highlights variability in sequence lengths, with the 2024 test set showing notably shorter sequences on average compared to other datasets. Regarding amino acid sequence length distributions (Figure 2b), the 2022 and 2024 test sets exhibit slightly lower amino acid frequencies, consistent with the smaller dataset sizes. Secondary structure frequency overviews for 3-state (Figure 2c) and 8-state classifications can be seen in Figure 2c and Figure 2d, respectively.

Our initial dataset, collected from all PDB entries available as of November 16, 2022, contained 500,624 protein sequences. In the first step, we used blast+(2.12.0+ds-3build1) [6] to perform 80% sequence identity clustering to select representative sequences, resulting in a dataset of 55,500 PDB sequences (PDB: 80%), among the largest used to build a secondary structures predictor. Subsequently, a more stringent filter was applied, reducing the dataset to 25,600 proteins at a 30% sequence identity threshold (PDB: 30%). Therefore, we divided PDB:30% into 1/5 for testing and 4/5 for training to conduct preliminary testing. As a reason for constructing datasets with different sequence identities, a larger dataset with a more significant number of protein sequences can introduce a higher level of diversity, therefore enriching the model training process. In addition, a secondary structure (SS) state was assigned based on the Dictionary of Protein Secondary Structures (DSSP) [19].

For testing purposes, we started using our primary testing 1/5 from PDB: 30%. For final evaluations, we employed a test set constructed from PDB entries released between 16 November 2022 and 20 July 2024. This dataset was redundancy-reduced at a 30% sequence identity threshold against the training set and internally to eliminate redundancy. Lastly, all the proteins with at least 10 undetermined amino acids (AA) were deleted from both datasets. The 2024 test set was reserved for the final phase of this study to benchmark our final model against previously published solutions.

To robustly assess the performance of our protein secondary structure prediction (PSSP) model, we conducted a 5-fold cross-validation using two clustered datasets: PDB: 80% and PDB: 30%. The PDB: 80% dataset, consisting of 55,500 sequences clustered at an 80% sequence identity threshold, was used as the training set across all folds. The PDB: 30% dataset, comprising 25,600 sequences clustered at a more stringent 30% sequence identity threshold, was divided into five equal parts containing approximately 5120 sequences, each serving as a test set in one of the five cross-validation folds. For each fold, the sequences in the test set (one part of PDB: 30%) were compared against the PDB: 80% dataset using MMseqs2 (13-45111+ds-2) [20] with a 30% sequence identity threshold. Sequences from PDB: 80% that did not match any sequence in the test set above the 30% identity threshold were selected to form the training set for that fold, to ensure that no significant sequence identity existed between training and testing data, thus preserving the integrity of the evaluation.

Additionally, the 2024 test set was utilized in the final phase of the study to perform a comparative evaluation of our final model against other solutions reported in the literature. This rigorous approach to dataset selection, training, and evaluation ensures that our PSSP model is both robust and generalizable, capable of achieving high accuracy across diverse protein sequences.

### 4.2. Sequence Embedding

This study explores various embedding techniques (Table 10), including the foundational one-hot encoding and advanced protein language model (PLM)-based methods such as ProtTrans and ESM-2. For classification tasks, our paradigms are utilized: a streamlined 3-class system, predicting with the 3-class model, the classes are harmoniously merged. Here is how: H, G, and I collectively represent the “helix” class; E and B seamlessly integrate to become the “strand” class; while S, T, and C unite under the “coil” designation.

#### 4.2.1. One-Hot Encoding

One-hot encoding is a foundational technique representing each amino acid with a 21-element vector. This vector captures the inherent structure of the sequence, where each amino acid is uniquely identified by a matrix indicating its presence (1) or absence (0). This method ensures that the distinct identity of each amino acid is preserved within the sequence representation.

#### 4.2.2. PLM-Based Embedding

PLM-based embedding, exemplified by ProtTrans and ESM-2, harnesses pre-trained language models to transmute protein sequences into vectorized representations. ProtTrans, trained on the UniRef50 dataset, generates 1024-dimensional embeddings that capture intricate relationships between amino acids, offering a deep understanding of sequence properties. Additionally, we use the ESM-2 [19] model, which is trained on UniRef90 dataset and contains 650 million parameters. The embeddings generated by ESM-2 contain a range of information related to proteins, covering aspects from similarities to biochemical characteristics. Similar to the ProtTrans method, we start with the amino acid sequence as the input. This process produces an output matrix with dimensions based on the sequence length ×1280. Consequently, This means that each amino acid is represented in a 1280-dimensional vector space. However, the ESM-2 model exhibits a constraint, limiting its encoding capabilities to sequences with a maximum length of 1022 amino acids. With a maximum length of 1022 amino acids, both methods offer rich representations suitable for protein structure prediction.

### 4.3. Neural Network Architecture

At the initial stage, a feedforward neural network (FFNN) is used to construct a baseline model structure and predictor. The idea of this baseline model is that this model structure will only look at a single residue to perform prediction at a position and will not consider any context. To imply such an FFNN architecture, we chose to use a convolutional neural network (CNN) with kernel size 1 and 1 channel, and sigmoid as the activation function. The reason to use a CNN is that it can deal with variable lengths of inputs. This feature is very useful for dealing with protein sequences, since their lengths can be changed.

Beyond the baseline model, we explore more advanced architectures such as recurrent neural networks (RNNs), long short-term memory (LSTM) networks, CNNs, and convolutional bidirectional recurrent neural networks (CBRCNNs) to assess their suitability for protein secondary structure prediction.

RNNs and LSTMs are particularly well-suited for capturing long-range dependencies in sequential data, which is critical for understanding the interactions between amino acids that are far apart in a protein sequence. CNNs, while originally designed for spatial data, are adapted here to capture local patterns in sequences through convolutional and pooling layers.

A key model variation we explore is the CBRCNN, which integrates bidirectional recurrent neural networks (BRNNs) with convolutional neural networks (CNNs). This hybrid architecture, referred to as CBRNN, is designed to leverage both sequence-wide dependencies and local motifs within protein sequences, combining the strengths of both RNN and CNN architectures.

In the CBRNN model, the BRNN component processes protein sequences in both forward and backward directions, capturing contextual information from the entire sequence. This bidirectional processing is crucial for understanding interactions between amino acids that may be distant from each other in the sequence, thereby enhancing the accuracy of secondary structure predictions.

Following the BRNN layers, convolutional layers are applied to further process the sequence representations. These layers are adept at identifying local sequence patterns, such as helices and strands, which are essential for accurate structural predictions. The convolutional layers apply a series of filters to the sequence data, extracting high-level features that are critical for the subsequent prediction tasks.

**Figure 3:**
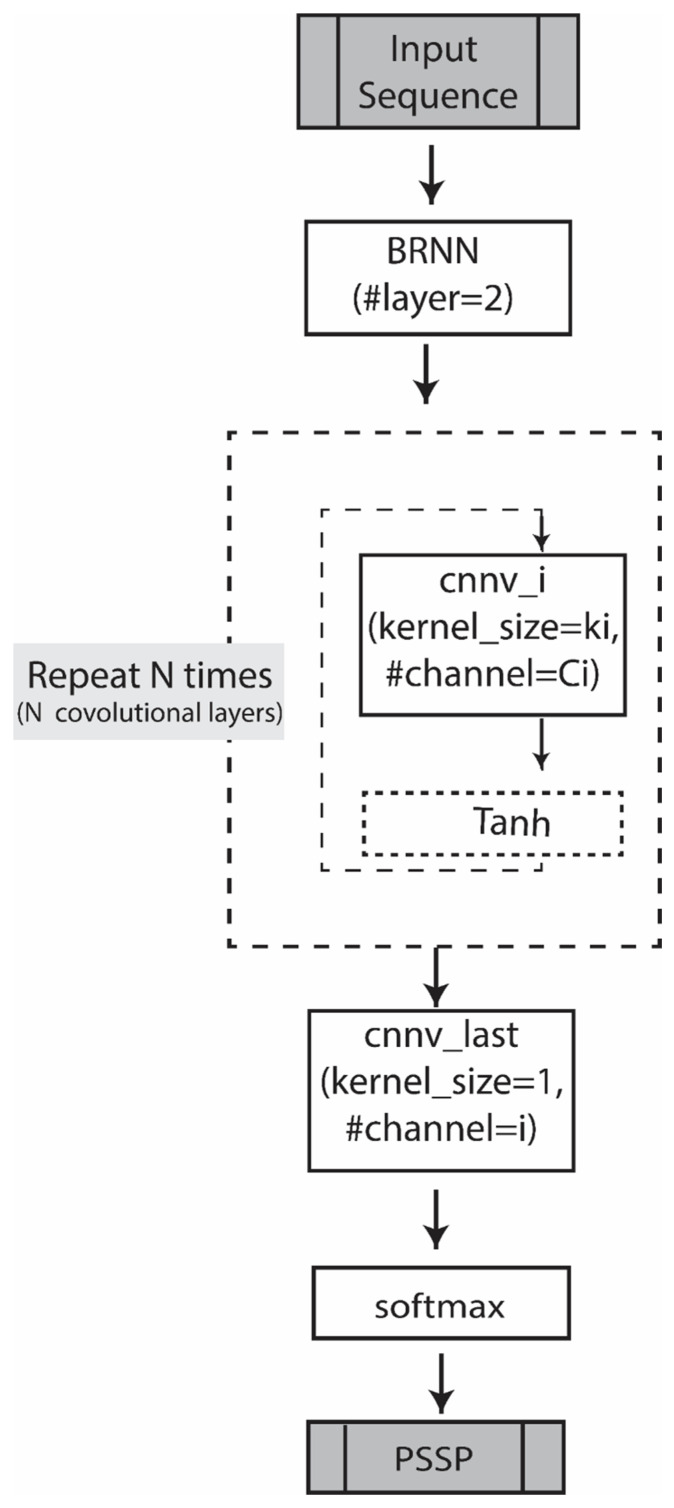
CBRNN structure for PSSP prediction, where N represents the total number of convolutional layers and i is the ith convolutional layer.

By integrating BRNN and CNN layers, the CBRNN model effectively combines the strengths of both architectures, enabling a more comprehensive analysis of protein sequences. This hybrid design enhances the model’s ability to accurately predict secondary structures by capturing both global contextual information and local sequence features. As shown in Figure 3, following the BRNN layers, convolutional layers are applied in a repeated structure to further process the sequence representations. Each convolutional layer (cnnv_i) applies filters with varying kernel sizes (ki) and channels (Ci), extracting local sequence patterns such as helices and strands. The final convolutional layer (cnnv_last) produces an output with the number of channels equal to the number of prediction classes (e.g., 3 for 3-class predictions or 8 for 8-class predictions). This output is then passed through a SoftMax activation function to generate class probabilities for each position in the sequence.

Table A1 outlines the architecture of CBRNN_L2, which was used to train on the PDB:30% dataset for 3-class predictions. The architecture includes 2 bidirectional BRNN layers with a hidden size of 40 and a CNN component consisting of 2 layers with varying kernel sizes, strides, and paddings.

For the larger PDB: 80% dataset, the architecture was optimized to improve performance for 3-class predictions, resulting in CBRNN_L3, which features 2 BRNN layers and 3 CNN layers, as detailed in Table A2. Similarly, for 8-class predictions, the architecture was extended to CBRNN_L4, which incorporates 2 BRNN layers and 4 CNN layers, as outlined in Table A3.

To further enhance the model, we experimented with increasing the depth of the CNN component by adding more layers beyond 3 and 4 for the respective prediction tasks. However, we observed diminishing returns, with no significant improvement in accuracy. As a result, we fine-tuned the model until additional depth no longer yielded enhancements, settling on the configurations of CBRNN_L3 and CBRNN_L4 as the optimal architectures for their respective datasets and tasks.

### 4.4. Performance Evaluation

Our evaluation focused on two key metrics for protein secondary structure prediction: accuracy in three-state (Q3) and eight-state (Q8) classifications. These metrics are widely used to assess the effectiveness of secondary structure prediction models. Accuracy is defined as the fraction of amino acids (AAs) whose predicted secondary structure (SS) class matches the observed class, as determined by DSSP. For the three-state classification problem (helix, sheet, and coil) this is referred to as Q3 accuracy [5]. For the more granular eight-state classification problem (α-helix, 3_10_-helix, π-helix, β-sheet, extended strand, hydrogen-bonded turn, bend, and other), it is termed Q8 accuracy [5].

To simplify the three-state classification, the eight DSSP-assigned secondary structure classes are grouped as follows: α-helix, 3_10_-helix, and π-helix are combined into the helix category; β-sheet and extended strand are merged into the sheet category; and all remaining classes are categorized as coil [5]. This approach ensures a consistent and interpretable comparison between Q3 and Q8 predictions while maintaining alignment with DSSP assignments.

## 5. Conclusions

This project aimed to develop a deep learning-based approach to protein secondary structure prediction (PSSP), leveraging modern protein language models (PLMs) and neural network architectures to achieve robust performance in predicting both three-state (Q3) and eight-state (Q8) secondary structures. This study utilized comprehensive datasets from the Protein Data Bank (PDB), filtered at 30% and 80% sequence identity thresholds, with an independent 2024 benchmark test set for final evaluation.

Our findings highlight the superiority of PLM-based embeddings, specifically ESM-2, which consistently outperformed traditional one-hot encoding methods and ProtTrans. By replacing multiple sequence alignment (MSA) techniques with PLM-based embeddings, we significantly reduced computational complexity without sacrificing accuracy. This suggests that PLMs can efficiently capture the necessary sequence information for PSSP, providing a scalable alternative to MSA-based methods.

In terms of model architecture, the convolutional bidirectional recurrent neural network (CBRNN) emerged as the most effective, especially when paired with deeper CNN layers. These architectures, trained on datasets with an 80% sequence identity threshold, delivered the highest Q3 and Q8 accuracy scores. The results indicate that using larger, diverse datasets can enrich model performance, though there may be diminishing returns with more stringent sequence identity thresholds.

The project compared Porter 6, the final model, against state-of-the-art predictors such as Porter 5, NetSurfP-2.0, NetSurfP-3.0, and SPOT-1D-LM. Porter 6 outperformed these models in both Q3 and Q8 accuracy, particularly on the 2024 benchmark set, confirming the efficacy of our approach. The exclusion of MSAs in place of PLMs was a key factor in improving both scalability and performance. Additionally, Porter 6 is limited to secondary structure prediction and relies on the quality of embeddings from protein language models, which may impact performance in some cases.

Ultimately, this work contributes a novel, efficient PSSP predictor that overcomes many limitations of previous models. Porter 6, publicly available for use and further development, offers a scalable solution for researchers in bioinformatics and protein structure analysis. Its success in handling large-scale datasets without the need for complex alignment processes demonstrates its potential for advancing research in areas such as drug discovery and biotechnology. Porter 6 is publicly available on GitHub (https://github.com/WafaAlanazi/Porter6 (accessed on 26 September 2024)).

## Figures and Tables

**Table 1:** Performance of various embedding methods using the FFNN model architecture.

Embedding	MAX-Length	ACC (Q3)
One-hot	-	66.00%
ProtTrans	-	85.53%
Esm-2	1024	85.68%

The ESM-2 model has a sequence length limitation of 1024 tokens, including the start and end tokens (i.e., 1022 sequence tokens).

**Table 2:** Performance of different embedding methods and architectures in Phase 1 (accuracy Q3).

Predictors	Embedding			ACC (Q3)
	One-Hot	ProtTrans	ESM-2	
CNN	1	0	0	71.69
	0	1	0	85.97
	0	0	1	86.22
BLSTM	1	0	0	70.07
	0	1	0	85.90
	0	0	1	86.10
BRNN	1	0	0	70.67
	0	1	0	85.97
	0	0	1	86.14
CBRNN	1	0	0	73.03
	0	1	0	85.99
	0	0	1	86.34

**Table 3:** CBRNN performance on three-class and eight-class predictions for PDB: 30% dataset.

Dataset	ACC (Q3)	ACC (Q8)
PDB: 30% (20K)	86.34%	75.23%

**Table 4:** Comparison of full-set training and five-fold cross-validation on the PDB: 30% dataset.

Training Strategy	ACC (Q3)	ACC (Q8)
Full set	86.34%	75.23%
Five-fold cross-validation	86.60%	75.43%

**Table 5:** Detailed performance of five-fold cross-validation for PDB: 30%.

Folds	ACC (Q3)	ACC (Q8)
Fold1	86.12%	74.86%
Fold2	86.10%	75.21%
Fold3	86.37%	75.10%
Fold4	86.31%	73.31%
Fold5	86.12%	74.99%
Average	86.20%	74.69%

**Table 6:** Detailed performance of five-fold cross-validation for PDB: 80%.

Folds	ACC (Q3)	ACC (Q8)
Fold1	86.47%	76.06%
Fold2	86.39%	75.17%
Fold3	86.44%	73.38%
Fold4	86.41%	75.32%
Fold5	86.61%	75.55%
Average	86.46%	75.10%

**Table 7:** Porter 6 on NMR vs. X-ray crystallography proteins in 2024 test set.

Method	ACC (Q3)	ACC (Q8)
X-ray	84.62%	74.26%
NMR	82.44%	67.87%

**Table 8:** Comparison of Porter 6 and state-of-the-art methods on the 2024 test set.

Method	ACC (Q3)	ACC (Q8)
Porter6 (PDB: 80%)	84.56%	74.18%
Porter6 (PDB: 30%)	84.40%	74.01%
Porter5	81.03%	70.03%
NetSurfP-2.0	81.37%	70.06%
NetSurfP-3.0	82.92%	71.84%
SPOT-1D-LM	84.30%	74.09%

**Table 9:** PSSP dataset information.

Data	Num of	Strategy
PDB 30% train	20,535	Blast 30% identity clustering
PDB 80% train	55,600	Blast 80% identity clustering
2022 test set	5130	Blast 30% identity clustering
2024 test set	692	Blast 30% identity clustering

**Table 10:** Information for the embedding methods.

Name	PLM	Embedding Dim
One-hot	20,535	21
ProtTrans	ProtT5-XL-UniRef50	1024
ESM-2	esm2-t33-650M-UR50D	1280

## Data Availability

Porter 6 is publicly available on GitHub (https://github.com/WafaAlanazi/Porter6 (accessed on 26 September 2024)).

## Appendix A

The model structures for CBRNN architectures are detailed below, each with specific configurations and hyperparameters.

CBRNN_L2: As shown in Table A1, the best models when trained on PDB: 30% for three classes.
CBRNN_L3: As shown in Table A2, when we use PDB: 80%, we increase the CNN layer to L3 for three classes.
CBRNN_L4: As shown in Table A3, when we use PDB: 80%, we increase the CNN layer to L4 for eight classes.

**Table A1:** Model structure of CBRNN_L2.

**BRNN Layer**						
**#layer**	**Layer Type**	**Input Size**	**Hidden Size**	**Output Size**	**Bidirectional**	**Number Layers**
1	RNN	#features	40	80 (40 × 2)	YES	2
**CNN Layer**						
**#Layer**	**#in Channel**	**#Out_Channel**	**Kernel Size**	**Stride**	**Padding**	**Activation Function**
1	80	10	7	1	3	Tanh
Last	10	3	1	1	0	SoftMax

Learning rate is 0.00005.

**Table A2:** Model structure of CBRNN_L3.

**BRNN Layer**						
**#Layer**	**Layer Type**	**Input Size**	**Hidden Size**	**Output Size**	**Bidirectional**	**Number Layers**
1	RNN	#features	40	80 (40 × 2)	YES	2
**CNN Layer**						
**#Layer**	**#in Channel**	**#Out_Channel**	**Kernel Size**	**Stride**	**Padding**	**Activation Function**
1	80	10	7	1	3	Tanh
2	10	8	3	1	1	Tanh
Last	8	3	1	1	0	SoftMax

Learning rate is 0.00005.

**Table A3:** Model structure of CBRNN_L4.

**BRNN Layer**						
**#Layer**	**Layer Type**	**Input Size**	**Hidden Size**	**Output Size**	**Bidirectional**	**Number Layers**
1	RNN	#features	40	80 (40 × 2)	YES	2
**CNN Layer**						
**#Layer**	**#in Channel**	**#Out_Channel**	**Kernel Size**	**Stride**	**Padding**	**Activation Function**
1	80	15	7	1	3	Tanh
2	15	10	3	1	1	Tanh
3	10	8	3	1	1	Tanh
Last	8	8	1	1	0	SoftMax

Learning rate is 0.00005.
